# The Role of Pharmaceutical Companies in Antimicrobial Stewardship: A Case Study

**DOI:** 10.1093/cid/ciaa053

**Published:** 2020-01-23

**Authors:** Elizabeth D Hermsen, Richard L Sibbel, Silas Holland

**Affiliations:** Merck & Co., Inc., Kenilworth, New Jersey, USA

**Keywords:** antimicrobial stewardship, antibiotic prescribing, antimicrobial resistance, One Health, industry

## Abstract

Rising levels of antimicrobial resistance pose serious dangers to patients, population health, food security, and economic stability worldwide. In response to this threat, the United Nations and the World Health Organization have called for multisectoral, multidisciplinary action, recognizing that human, animal, and environmental health are interdependent. Although the pharmaceutical industry clearly has a leading role in developing novel antimicrobials and vaccines, it is also active in many areas supporting antimicrobial stewardship. This article describes why pharmaceutical companies invest in antimicrobial stewardship, outlines why they are well suited to help address this issue, and provides examples of how the pharmaceutical industry can support the responsible use of antimicrobials. Merck & Co., Inc. (Kenilworth, NJ, USA), a large, globally operating pharmaceutical company that develops and markets both human and veterinary antimicrobials and vaccines is used as a case study for illustrating industry involvement in antimicrobial stewardship efforts.

## Teaser

Urgent multisectoral action is necessary to combat rising antimicrobial resistance. We explain why pharmaceutical companies invest in antimicrobial stewardship and why they are well suited to help address this issue, providing examples of how industry can support responsible antimicrobial usage.

**(See the Editorial Commentary by Tamma and Cosgrove on pages 682–4.)**

Rising levels of antimicrobial resistance (AMR) pose serious dangers to patients, population health, food security, and economic stability worldwide [1]. In response to this threat, the United Nations and the World Health Organization (WHO) have called for multisectoral, multidisciplinary action, recognizing that human, animal, and environmental health are interdependent [1]. The WHO Global AMR Action Plan and many national AMR action plans further underscore the need for multisector stakeholder cooperation, including governments, agriculture, healthcare providers, and the pharmaceutical industry. Although the pharmaceutical industry clearly has a leading role in developing novel antimicrobials and vaccines, it is also active in many areas supporting antimicrobial stewardship (AMS). Some companies have publicly committed to tackle AMR by promoting responsible antimicrobial usage [2, 3]. Here we describe why pharmaceutical companies invest in AMS, outline why they are well suited to help address this issue, and provide examples of how the pharmaceutical industry can support the responsible use of antimicrobials, using as an example, Merck & Co., Inc. (Kenilworth, NJ, USA; known as MSD outside the USA and Canada), a large, globally operating pharmaceutical company that develops and markets both human and veterinary antimicrobials and vaccines [2].

## WHY DO PHARMACEUTICAL COMPANIES INVEST IN AMS?

First and foremost, AMS is essential to improving patient outcomes and quality of care. Companies operating in the infectious diseases space have a social responsibility to support such stewardship. Although it may seem that companies’ commercial objectives could undermine responsible use of novel antibiotics, in fact AMS is an example of where “doing well” and “doing good” are not mutually exclusive. Supporting AMS presents opportunities to collaborate with key stakeholders on a public health priority to develop and deliver solutions that benefit both population health as well as commercial viability in 4 ways. One, improved surveillance and greater awareness about regional/local resistance patterns and the impact of AMR enables healthcare providers to recognize patient populations in whom specific antimicrobials are needed—this, in turn, can facilitate patient access to appropriate novel antimicrobials. Two, responsible use of novel antimicrobials, which leads to optimized patient outcomes, can foster advocacy for effective products among healthcare providers. Three, industry efforts to reduce inappropriate use of antimicrobials have the potential to slow the development of AMR and prolong the commercial lifespan of currently available antimicrobials. Four, AMS may help protect the long-term viability of product portfolios in other therapeutic areas. For example, drug-resistant infections in high-risk patients, such as oncology, diabetic, or surgical patients, may negatively impact treatment outcomes for these patients’ underlying conditions. Ultimately, strong investments in AMS should be part of the license to operate for companies active in the infectious diseases space.

## WHAT MAKES PHARMACEUTICAL COMPANIES WELL SUITED TO SUPPORT AMS?

Effective AMS relies on understanding human behavior, identifying areas of unmet need, communicating effectively, building advocacy, evaluating the most effective strategies and disseminating information on those strategies, and working as a team. Pharmaceutical companies, particularly large, global organizations, possess various capabilities consistent with these required skills. Specifically, such companies tend to have (*a*) employees with broad, multidisciplinary experience, including clinical practice, pharmacoeconomics, epidemiology, health literacy, communications, consumer behavior, market research and analytics, and marketing; (*b*) the ability to leverage various information-sharing platforms; (*c*) a broad customer network to facilitate sharing of best practices and how to overcome barriers; (*d*) a worldwide network of institutions with the necessary infrastructure for conducting clinical studies and surveillance; and (*e*) a global perspective coupled with an understanding of local needs. Although pharmaceutical companies do not have primary responsibility for the successful implementation of AMS programs, the skill sets found within pharmaceutical companies can effectively complement those of other stakeholders in multisector AMS collaborations.

## WHAT SPECIFIC ACTIONS CAN PHARMACEUTICAL COMPANIES TAKE TO SUPPORT AMS?

To describe the range of potential AMS activities, Merck & Co., Inc. can serve as an illustrative case study. The foundation of Merck & Co., Inc.’s overall AMS strategy is a One Health approach, that is, working with stakeholders in human health, animal health, and environmental sciences to achieve the best health outcomes for people, animals, and the environment [2]. Informed by this One Health perspective and underpinned by advocacy for policy solutions to address the global challenges limiting development of and access to new antibiotics, vaccines, and diagnostics needed to address AMR, Merck & Co., Inc. is active in AMS awareness and education, surveillance and research, implementation, and advocacy (Table 1). Notable examples of this work include:

**Table 1.** List of Potential Antimicrobial Stewardship Activities That Can be Supported by Pharmaceutical Industry, Using as a Case Study Merck & Co., Inc. (Kenilworth, NJ, USA), a Large, Multinational Company Marketing Antimicrobials and Vaccines for Human and Veterinary Use

Type of Activity	Example
**AMS awareness and education**	
AMS research methodology training	Independent educational grant for development of an annual conference focused on research methodology training and best-practice sharing for AMS clinicians, which was convened by the Society for Healthcare Epidemiology of America AMS each year from 2016 to 2018.
Online AMS resources	Independent grant for the Center for Infectious Disease Research and Policy’s Antimicrobial Stewardship Project, a multifunctional, online AMS resource providing comprehensive, high-quality information and interactive discussion. This content-rich website is designed to engage a diverse, international audience and includes a broad range of AMS-related topics, such as clinical practice, infection control/prevention, diagnostics, policy/legislation, research, public health, and veterinary medicine [9].
Patient engagement	Independent grant for George Washington University in collaboration with the Urgent Care Association of America to develop and implement an educational campaign to improve AMR and AMS health literacy and maintain or improve patient satisfaction in the urgent care clinic setting [10].
Patient advocacy	Financial support to the Peggy Lillis Foundation [11]. Additionally, health literacy expertise was utilized to redesign an educational brochure on *Clostridioides difficile* infection for the Foundation [12].
Veterinary educational campaigns	Program (“Time to Vaccinate”) that advises farmers how to keep ruminant herds healthy and productive by increasing immunity against various pathogens through vaccination and improved housing, hygiene, and nutrition; fewer outbreaks of livestock infectious diseases help reduce the need for antimicrobial use in agriculture.
Raise awareness in the private sector	Signatory to the Global Chief Medical Officer’s Network pledge on AMR, which states that each signatory company commits to promoting AMS among their employees, including employee education and tracking and reporting the company’s AMS initiatives [13].
Promotional practices	(1) Training on AMS principles for sales representatives and all other relevant field staff (in both human and animal health), in order to encourage responsible use of antimicrobials. (2) Pilot program in which field sales representatives’ performance incentives are not based on antimicrobial sales volume.(3) Regular assessment of all promotional materials to ensure they encourage responsible use of antimicrobial agents; this assessment utilizes a stewardship framework referred to as the “Star of Stewardship” [14].
**AMR surveillance and AMS research**	
Global surveillance programs	SMART global surveillance program, in which isolates from various bacterial infection types are collected and tested at a central laboratory for susceptibility to the company’s antibacterial agents, as well as to other, widely used agents.
Local surveillance programs	Generation of local susceptibility data, including technical assistance with the evaluation of local epidemiology and antibiogram development.
Animal health surveillance	Commercial surveillance partnership with Kansas State University for monitoring multidrug-resistant bacteria across the live animal, beef, and dairy production chain.
Investigator-initiated AMS research	Investigator-initiated research studies around the world, with specific areas of interest related to AMS, which are defined and released every year [15].
Funding not-for-profit vaccine research	Joint-venture partnership with the Wellcome Trust to fund and support Hilleman Laboratories, a not-for-profit organization aiming to create new vaccines and improved vaccine technology for diseases predominantly affecting low-income countries [16].
**AMS implementation**	
Reduce environmental impact from production of antibiotics	Commitment of $100 million to a water-infrastructure improvement initiative to install active pharmaceutical ingredient-treatment technology at all antibiotic manufacturing facilities.
Facilitate patient-centered AMS program implementation in acute care hospitals	(1) Support a collaborative project with the U.S. CDC and the Duke Antimicrobial Stewardship Outreach Network to develop practical and meaningful safety outcome measures for hospital AMS programs and to provide a publicly available guide to help hospitals collect and report such outcomes [17]. (2) Support the *Antibiotic Stewardship in Acute Care Playbook*, published by the National Quality Forum; this is a manual providing practical guidance for acute care facilities on how to implement the U.S. CDC’s core elements of hospital AMS programs [18]. (3) Serve as a knowledge, resource, and/or logistics partner to hospitals outside the United States to assist with antibiogram and clinical pathway development, education of providers, and monitoring outcomes [19]. (4) Establish AMS Centers of Excellence via partnerships with various organizations, whereby the partner organization serves as a central hub within their region to provide training for and assistance with hospital AMS program implementation [6, 7].
Support for development and implementation of national AMR and AMS action plans	(1) Independent grant to the Center for Disease Dynamics, Economics, and Policy’s Global Antibiotic Resistance Partnership to develop actionable, country-level AMR plans for low- and middle-income countries [20]. (2) Independent grant to the Pan-American Health Organization and Florida International University to develop regional AMS guidelines for Latin America and the Caribbean, resulting in a subsequent guideline publication [21].
Decision support tools	(1) Commercially available technology, through a subsidiary, designed to help health systems address the unique challenges of infectious diseases, with the goals of helping hospitals accelerate patient access to appropriate interventions, efficiently track prescribing and patient outcomes, and communicate within the hospital workflow [22]. (2) “Poultry Convenience” program provides veterinarians and farmers with technical support and expertise to help protect poultry against major respiratory and immune diseases, including a component on achieving optimal animal vaccination and welfare standards. (3) A web- and mobile phone app-based management system that helps individual farms reduce the impact of the porcine reproductive and respiratory syndrome disease complex through appropriate control and prevention strategies, with a focus on optimizing vaccination. (4) “BeSecure” program improves internal and external biosecurity, thus reducing pathogen entry into and circulation within farms.
Improved diagnostics	(1) Collaboration with OpGen to create diagnostic tests that can rapidly detect resistant infections and build a genomic knowledge base of antibiotic-resistant pathogens for predicting antibiotic susceptibility [23]. (2) Collaborations with antimicrobial susceptibility testing device manufacturers to expedite the inclusion of novel antibacterial agents on susceptibility testing panels. (3) Animal health division markets a stethoscope system that assists in a more accurate diagnosis of bovine respiratory disease, thus helping determine which animals require antibacterial treatment.
**AMS advocacy**	
Initiatives to advance policy supportive of AMS	Advocacy with infectious disease societies, patient groups, industry, and other partners to advance reimbursement reform proposals that enable appropriate patient access to novel antibiotics, including the DISARM Act in the United States and the Novel Antibiotic Subscription Model in the United Kingdom.
Initiatives to improve manufacturing practices	(1) Work to broaden adoption of the “Common Antibiotic Manufacturing Framework” across the industry [24]. (2) Work with third parties across the manufacturing and supply chain to ensure good practices for eliminating antibiotic discharge.

Abbreviations: AMR, antimicrobial resistance; AMS, antimicrobial stewardship; CDC, Centers for Disease Control and Prevention; SMART, Study for Monitoring Antimicrobial Resistance Trends.

- *Awareness and education:* The Merck Animal Health “Time to vaccinate” program is an initiative intended to accelerate the transition to vaccination becoming a more widely adopted preventative measure on farms as an immune health driver [4]. The educational resources, with content tailored to participating countries, advise farmers how to keep ruminant herds healthy and productive by increasing immunity against various pathogens through vaccination and improved housing, hygiene, and nutrition. The program intends to reduce outbreaks of livestock infectious diseases, therefore reducing the need for antimicrobial use in agriculture.
- *Surveillance and research:* Merck & Co., Inc. supports the Study for Monitoring Antimicrobial Resistance Trends (SMART), in which certain isolates from urinary tract, intra-abdominal, blood stream, and respiratory tract infections are collected and tested at a central laboratory for susceptibility to the company’s and numerous other, widely used antibacterial agents; isolates resistant to the tested antibiotic agents are molecularly characterized to provide comprehensive molecular epidemiology [5]. In an additional collaboration, Merck & Co., Inc. provides access to its archive of over 200 000 bacterial isolates gathered in the SMART program to its partner company OpGen, with the goals of building a genomic knowledge base of antibiotic-resistant pathogens and subsequently developing rapid DNA tests for predicting antibiotic susceptibility. Other pharmaceutical companies fund their own global surveillance studies, such as Pfizer’s ATLAS and the long-standing SENTRY Antimicrobial Surveillance Program supported by several industry sources. These programs are useful for identifying resistance trends over time, alerting the healthcare community to emerging resistance, and informing treatment decisions based on local/regional epidemiology.
- *Implementation:* Merck & Co., Inc. also collaborates with hospitals around the world to support development and implementation of patient-centered, product-agnostic AMS programs. This initiative began in 2008 in India but has since expanded to over 1100 hospitals in 28 countries. One approach is to work directly with individual institutions to provide education and resources in order to help them develop antibiograms, clinical pathways, and other components of AMS programs. Another approach is to work with a partner organization to establish a regional AMS Center of Excellence, which then provides AMS implementation support to institutions across the specific region. Recently published data from a partnership with the independent, nonprofit research institute CIDEIM show that the establishment of such regional centers results in improved adherence to antibiotic prescribing guidelines, reduced resistance rates, and healthcare cost savings [6, 7]. So far as part of this initiative, more than 10 000 healthcare providers have been trained by company-employed medical science liaisons (MSLs) and/or external scientific experts, and over 500 locally tailored clinical treatment pathways have been implemented.
- *Advocacy:* Through the AMR Industry Alliance, Merck & Co., Inc. is actively advocating to broaden adoption of the Common Antibiotic Manufacturing Framework; this is a set of minimum environmental expectations for antibiotic manufacturers, based on science-driven, risk-based target concentrations for managing antibiotic discharge from manufacturing operations [8]. Merck & Co., Inc. itself is investing heavily into installation of active pharmaceutical ingredient-treatment technology at antibiotic manufacturing facilities. Furthermore, the company is working with third parties across the manufacturing and supply chain to ensure good practices for eliminating antibiotic discharge.

Tackling the worldwide threat of AMR [1] will require action by a wide range of actors. Additional research is needed to evaluate the impact of industry-supported AMS efforts, particularly AMS education, patient engagement/advocacy, and novel reimbursement and sales incentive models, on outcomes and prescribing patterns. As illustrated by this case study, the pharmaceutical industry is well positioned to play a part in the global response—not only through drug and vaccine development but also through taking tangible action to support responsible antimicrobial use.

## Notes

***Author contributions.*** All authors contributed to the initial draft of the paper, critically reviewed and revised subsequent drafts for important intellectual content, and approved the final version for submission.

***Acknowledgments.*** Medical writing assistance was provided by Dominik Wolf, MS, and editorial assistance was provided by Michele McColgan, BA, both of Merck Sharp & Dohme Corp., a subsidiary of Merck & Co., Inc., Kenilworth, NJ, USA.

***Potential conflicts of interest.*** E. D. H. and S. H. are employees of Merck Sharp & Dohme Corp., a subsidiary of Merck & Co., Inc., Kenilworth, NJ, USA, and may own stock or stock options in the Company. R. L. S. is a former employee of Merck Sharp & Dohme Corp., a subsidiary of Merck & Co., Inc., Kenilworth, NJ, USA, and currently is a consultant to Merck Animal Health. All authors have submitted the ICMJE Form for Disclosure of Potential Conflicts of Interest. Conflicts that the editors consider relevant to the content of the manuscript have been disclosed.

## References

[1] World Health Organization. Global action plan on antimicrobial resistance. Geneva, Switzerland: World Health Organization, 2015. 10.7196/samj.9644 26242647

[2] Global antimicrobial resistance action plan. Merck & Co., Inc Available at: https://www.merck.com/about/views-and-positions/public-policy-statement-Global-Antimicrobial-Resistance-Action-Plan.pdf. Accessed 30 October 2018.

[3] Industry roadmap for progress on combating antimicrobial resistance. IFPMA, 2016 Available at: https://www.ifpma.org/resource-centre/industry-roadmap-for-progress-on-combating-antimicrobial-resistance/. Accessed 30 October 2018.

[4] Time to vaccinate. MSD Animal Health; 2018 Available at: https://www.timetovaccinate.com/. Accessed 14 November 2018.

[5] Morrissey I, Hackel M, Badal R, Bouchillon S, Hawser S, Biedenbach D A review of ten years of the study for monitoring antimicrobial resistance trends (SMART) from 2002 to 2011. Pharmaceuticals (Basel) 2013; 6:1335–46. 24287460 10.3390/ph6111335 PMC3854014

[6] Feinstein MM, Escandón-Vargas K, Reyes S, Hernández-Gómez C, Pallares CJ, Villegas MV Improved outcomes when antibiotic prescribing guidelines are followed by healthcare providers: a Colombian example to encourage adherence in hospital settings. Infect Control Hosp Epidemiol 2017; 38:756–8. 28366177 10.1017/ice.2017.45

[7] Hernández-Gómez C, Pallares C, Reyes S, et al Clinical impact of antimicrobial stewardship programs in Colombian acute care hospitals. Open Forum Infect Dis 2017; 4(suppl_1):S486–S7.

[8] Tell J, Caldwell DJ, Häner A, et al. Science-based targets for antibiotics in receiving waters from pharmaceutical manufacturing operations. Integr Environ Assess Manag 2019; 15:312–9. 30884149 10.1002/ieam.4141 PMC6849714

[9] Center for Infectious Disease Research and Policy. Antimicrobial stewardship project Available at: http://cidrap.umn.edu/asp. Accessed 13 October 2019.

[10] Antibiotics and you. Available at: www.antibioticsandyou.org. Accessed 29 January 2020.

[11] Peggy Lillis Foundation. About PLF funders Available at: https://peggyfoundation.org/about-plf/funders/. Accessed 13 October 2019.

[12] Hermsen ED, MacGeorge EL, Andresen ML, et al Decreasing the Peril of Antimicrobial Resistance Through Enhanced Health Literacy in Outpatient Settings: An Underrecognized Approach to Advance Antimicrobial Stewardship. Adv Ther. 2020 Jan 17. [Epub ahead of print] 10.1007/s12325-019-01203-1 PMC6999167 31953805

[13] Bupa. Antibiotic resistance: CMOs sign pledge encouraging appropriate use of antibiotics Available at: www.bupa.com/sharedcontent/articles/cmo-antibiotic-resistance. Accessed 13 October 2019.

[14] Merck & Co, Inc. Merck’s commitment to antimicrobial stewardship Available at: https://www.merck.com/FlipBook/GARAP/index.html#p=16. Accessed 13 October 2019.

[15] Investigator Studies Program (MISP): Antimicrobial Stewardship Available at: http://engagezone.msd.com/Antimicrobial_Stewardship.php. Accessed 13 October 2019.

[16] MSD Wellcome Trust Hilleman Laboratories Available at: https://hillemanlabs.org. Accessed 13 October 2019.

[17] Duke Antimicrobial Stewardship Outreach Network. Developing Stewardship Measures. Developing Patient Safety Outcome Measures and Measurement Tools for Antibiotic Stewardship Programs Available at: https://dason.medicine.duke.edu/developing-stewardship-measures. Accessed 13 October 2019.

[18] National Quality Forum Available at: www.qualityforum.org/Publications/2016/05/National_Quality_Partners_Playbook__Antibiotic_Stewardship_in_Acute_Care.aspx. Accessed 13 October 2019.

[19] Merck & Co, Inc. Merck’s commitment to antimicrobial stewardship Available at: https://www.merck.com/FlipBook/GARAP/index.html#p=10. Accessed 13 October 2019.

[20] The Center For Disease Dynamics, Economics & Policy. Global Antibiotic Resistance Partnership (GARP) Available at: https://cddep.org/partners/global-antibiotic-resistance-partnership/. Accessed 13 October 2019.

[21] Pan American Health Organization. New PAHO manual guides management of antimicrobial resistance in the Americas Available at: https://www.paho.org/hq/index.php?option=com_content&view=article&id=14804:new-paho-manual-guides-management-of-antimicrobial-resistance-in-the-americas&Itemid=1926&lang=en. Accessed 13 October 2019.

[22] Ilúm Health Solutions Available at: https://ilumhealthsolutions.com/ilum-home/. Accessed 13 October 2019.

[23] OpGen collaborates with Merck to develop novel rapid diagnostics and informatics tools to combat antibiotic resistance Available at: https://globenewswire.com/news-release/2016/11/14/889536/0/en/OpGen-Collaborates-With-Merck-to-Develop-Novel-Rapid-Diagnostics-and-Informatics-Tools-to-Combat-Antibiotic-Resistance.html. Accessed 13 October 2019.

[24] AMR Industry Alliance. Responsible manufacturing Available at: https://www.amrindustryalliance.org/shared-goals/common-antibiotic-manufacturing-framework/. Accessed 13 October 2019.

